# Novel Application of ^18^F-NaF PET/CT Imaging for Evaluation of Active Bone Remodeling in Diabetic Patients With Charcot Neuropathy: A Proof-of-Concept Report

**DOI:** 10.3389/fmed.2022.795925

**Published:** 2022-02-18

**Authors:** Nguyen K. Tram, Ting-Heng Chou, Surina Patel, Laila N. Ettefagh, Michael R. Go, Said A. Atway, Mitchel R. Stacy

**Affiliations:** ^1^Center for Regenerative Medicine, The Research Institute at Nationwide Children's Hospital, Columbus, OH, United States; ^2^Division of Vascular Diseases and Surgery, Department of Surgery, The Ohio State University College of Medicine, Columbus, OH, United States; ^3^Department of Orthopaedics, The Ohio State University College of Medicine, Columbus, OH, United States

**Keywords:** sodium fluoride, positron emission tomography, charcot, computed tomography, bone remodeling

## Abstract

Charcot neuropathic osteoarthropathy (CN) is a serious and potentially limb-threatening complication for patients with diabetes mellitus and peripheral arterial disease. In recent decades, nuclear medicine-based approaches have been used for non-invasive detection of CN; however, to date, a positron emission tomography (PET) radionuclide specifically focused on targeted imaging of active bone remodeling has not been explored or validated for patients with CN. The radionuclide ^18^F-sodium fluoride (NaF) has historically been used as a bone imaging probe due to its high sensitivity for targeting hydroxyapatite and bone turnover, but has not been applied in the context of CN. Therefore, the present study focused on novel application of ^18^F-NaF PET/computed tomography (CT) imaging to three clinical cases of CN to evaluate active bone remodeling at various time courses of CN. PET/CT imaging in all 3 cases demonstrated focal uptake of ^18^F-NaF in the bones of the feet afflicted with CN, with bone retention of ^18^F-NaF persisting for up to 5 years following surgical reconstruction of the foot in two cases. On a group level, ^18^F-NaF bone uptake in the CN foot was significantly higher compared to the healthy, non-CN foot (*p* = 0.039). ^18^F-NaF PET/CT imaging may provide a non-invasive tool for monitoring active bone remodeling in the setting of CN, thereby offering novel opportunities for tracking disease progression and improving treatment and surgical intervention.

## Introduction

Charcot neuropathic osteoarthropathy (CN) is a condition that can impair quality of life and increase risk of limb loss in patients with diabetes mellitus (DM) and peripheral arterial disease (PAD) (1, 2). CN is characterized by local inflammation in the early phase of the condition, followed by degeneration of the bone architecture and ulceration of soft tissues in the foot and ankle in the later phases (3). If left untreated, CN can result in major disruption of normal skeletal structure that can cause significant loss of function and increased morbidity (4). Current treatment for early phases of CN consists of offloading the affected foot using a total contact cast (5), while common surgical interventions for later stages of CN include exostectomy, application of fixators, and minor or major amputations. The overall rate of amputation in patients with CN and diabetes is 3.3–11 per 1,000 patients, with 70–84% of these patients having a preceding ulceration (6). Patients with CN and diabetes undergoing amputation also have a high mortality rate that is ~70% in the first 5 years after amputation (7).

Imaging techniques such as radiography and magnetic resonance imaging (MRI) are often used to evaluate CN. However, x-ray imaging possesses low sensitivity and specificity for diagnosing the early stages of CN (8) while MRI can poorly differentiate between CN and osteomyelitis, which often exist concurrently (9). The nuclear imaging-based approaches of scintigraphy and single photon emission computed tomography (SPECT) are commonly used to detect the early stages of inflammation that precede bone morphology changes related to CN and ultimately assist with early diagnosis of CN. One traditional scintigraphy/SPECT radionuclide used for diagnosing CN is technetium-99m (^99m^Tc)-methylene diphosphonate (MDP), a bone targeted radionuclide which possesses excellent sensitivity for diagnosing CN and can assist with differentiating between osteomyelitis and CN (10). Another scintigraphy- and SPECT-based approach that has been used for imaging of CN includes the use of ^99m^Tc-MDP bone imaging with indium-111 (^111^In)-labeled white blood cells (WBC) or ^99m^Tc-WBC (11–13), which has been shown to be useful for distinguishing between soft tissue vs. bone infection in patients with CN. While these imaging methods have proven useful, the current gold standard for differentiating between foot infection vs. CN remains dual-isotope scintigraphy/SPECT imaging with ^99m^Tc-sulfur colloid and ^111^In-WBC, which possesses the best accuracy for detecting CN (14–16).

Along with scintigraphy and SPECT imaging methods, PET imaging with fluorine-18 (^18^F)-fluorodeoxyglucose (FDG) has emerged in recent years for evaluating the inflammatory origins of CN (17). Multimodality imaging studies have demonstrated superior accuracy of ^18^F-FDG PET vs. MRI in the diagnosis of CN lesions (17, 18) and higher specificity of PET/CT than MRI for diagnosing osteomyelitis in patients with chronic CN (19). Additionally, ^18^F-FDG PET/CT imaging has shown potential for monitoring of serial changes in inflammation in patients with CN (20).

While SPECT- and PET-based imaging approaches have shown promise for evaluating CN, a PET imaging method that specifically targets active bone remodeling in the setting of CN has not been investigated, which would offer considerable advantages over current SPECT methods by providing improved image spatial resolution and quantification. ^18^F-sodium fluoride (NaF) has historically been used since the 1960s as a radionuclide for targeting bone remodeling due to its high affinity for hydroxyapatite, the mineral form of calcium apatite (21). ^18^F-NaF has also been used for other indications, such as low back pain (22), brown tumors in hyperparathyroidism (23), and bone metastases (24). However, to date, ^18^F-NaF has not been studied in the context of CN. Additionally, while several imaging approaches have been used to identify early onset CN (stage 0) (25), targeted bone imaging in patients with CN following surgical intervention remains understudied. Therefore, the purpose of this study was to evaluate the utility of ^18^F-NaF PET/CT imaging as a tool for non-invasively characterizing active bone remodeling in a series of patients with CN who were at various time points following surgical reconstruction of the foot.

## Methods

Three patients with CN, type 2 diabetes mellitus (DM), and peripheral arterial disease (PAD) were prospectively enrolled into an ongoing study evaluating the prognostic value of nuclear imaging techniques in patients with PAD (https://clinicaltrials.gov, NCT03622359) (26). As an additional component of this study, PET/CT imaging was performed 75 min after intravenous injection of ^18^F-NaF (375.6 ± 10.9 MBq) to evaluate active remodeling of the bones in the feet. All patients underwent PET imaging using a commercially available scanner (Discovery PET/CT 690, GE Healthcare). A low-dose CT scan of the feet was also acquired to guide manual image segmentation of the bones in the feet and ankles, and for PET image attenuation correction. All PET data was converted to standardized uptake values (SUVs) following correction for injected dose, patient body weight, attenuation, and radionuclide decay.

Semiautomated segmentation of the bones of the ankle and foot from CT images for each limb was performed using commercially available image analysis software (PMOD Technologies LLC, Zürich, Switzerland). First, a volume of interest (VOI) was drawn around the foot. Second, within the foot VOI, any tissues with Hounsfield units (HUs) equal to or >100 HU were classified as bone, based on our own experience evaluating common HU values for bones of the feet in our clinical sample. Following segmentation of bones using this approach, the segmentation was further evaluated for accuracy and manually corrected on a slice-by-slice basis, as needed.

For PET image analysis, the average target-to-background ratio (TBR_avg_) of each foot was calculated using the SUVs acquired for each leg, with the average SUV across all bones of the feet representing the target value while a small (5 mm^3^) piece of the cortical bone of the tibia represented the background value. A paired *t*-test was used to compare the TBR between the diseased foot and the healthy foot. A *p* < 0.05 was considered statistically significant. All statistical analysis was performed using Prism v9 for macOS (GraphPad Software, San Diego, CA, USA).

## Results

**Figure 1:**
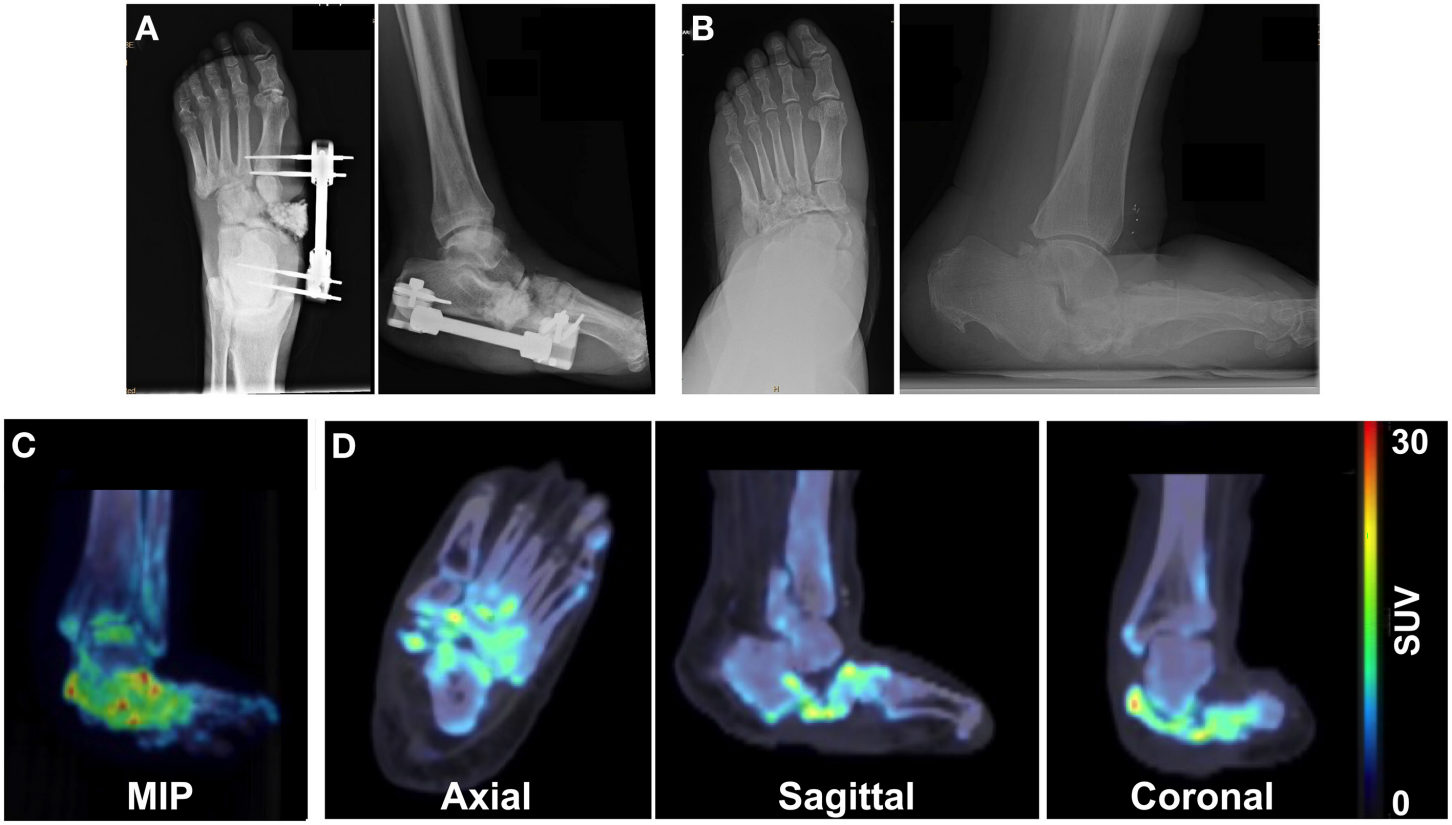
Multimodality imaging evaluation of a 65-year old male patient with a history of Charcot foot, type 2 DM, and PAD. X-rays acquired at **(A)** the time of external fixation and **(B)** 5 years after surgery reveal the architecture of the foot. **(C)** Maximum intensity projection (MIP) images and **(D)** axial, sagittal, and coronal ^18^F-NaF PET/CT images of the foot 5 years after surgery demonstrate focal increased uptake of ^18^F-NaF in the bones of the foot, indicating ongoing physiological remodeling of the afflicted foot 5 years after surgical reconstruction.

Patient 1 initially presented with a chronic left foot wound deep to the level of bone. MRI was consistent with osteomyelitis of the navicular bone as well as the medial and middle cuneiform bones. The patient also had a history of CN due to type 2 DM. Conservative treatment options were exhausted, and the patient underwent surgical intervention. External fixation was applied to the medial foot, which consisted of a Biomet Mini-Rail and four half pins across the mid-tarsal joint (Figure 1A) that were then removed 2 months later. Five years after surgery, radiography demonstrated stable bone structures (Figure 1B). PET/CT images were also acquired 5 years after surgery and revealed focal uptake of ^18^F-NaF within the bones of the foot with CN, which suggested ongoing bone remodeling 5 years post-surgical reconstruction (Figures 1C,D).

**Figure 2:**
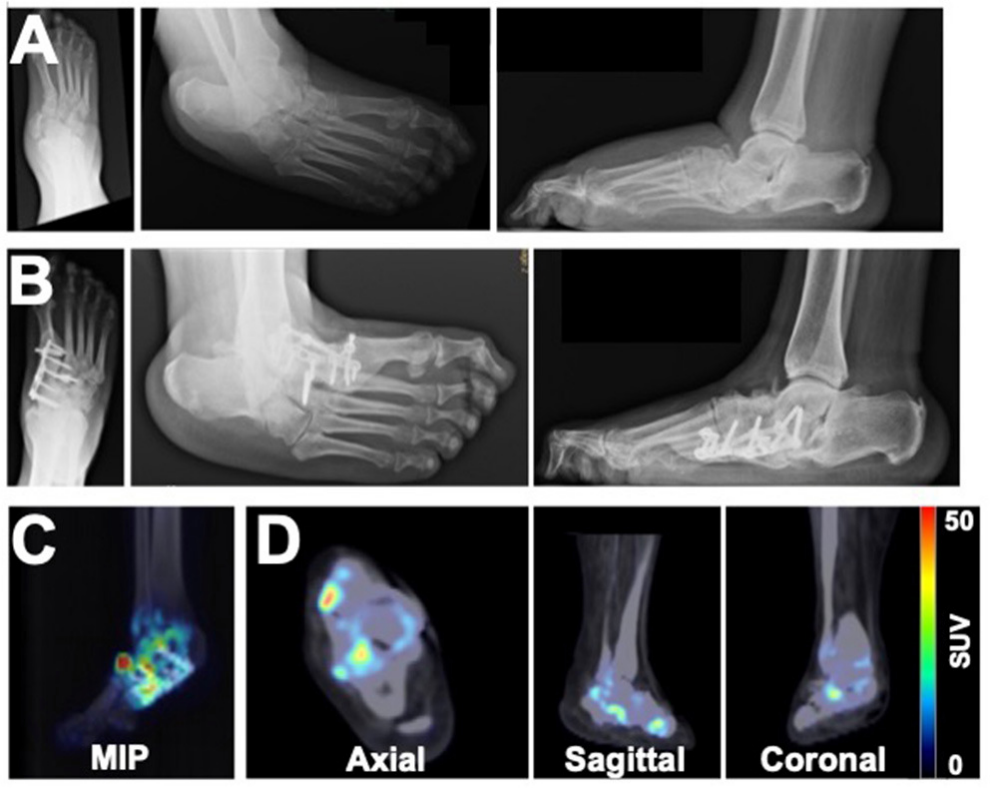
Imaging assessment of a 49-year old female patient with a history of right foot Charcot deformity. X-rays were acquired at **(A)** the time of navicular bone removal and **(B)** 5 years after surgical reconstruction of the foot. **(C)** MIP and **(D)** PET/CT images acquired 5 years after surgery revealed heterogeneous boney uptake of ^18^F-NaF and suggested potential for ongoing remodeling of the foot.

Patient 2 originally presented with a Charcot deformity of the right foot with chronic ulceration to the medial aspect of the foot. Following successful wound healing, the patient elected to have surgical intervention to reconstruct the foot and excise the prominent navicular bone (Figure 2A). After complete removal of the navicular bone, a medial column BioMet Advanced Locking Plate System plate was fixated across the talo-medial cuneiform joint and across the first tarsometatarsal joints using a total of three non-locking and four locking screws. Five years after surgery, x-rays demonstrated stable bone architecture with partial fusion at the midfoot architecture (Figure 2B). However, PET/CT images also acquired 5 years post-surgery revealed increased focal uptake of ^18^F-NaF in the bones of the right foot, thus indicating an ongoing process of bone remodeling (Figures 2C,D).

**Figure 3:**
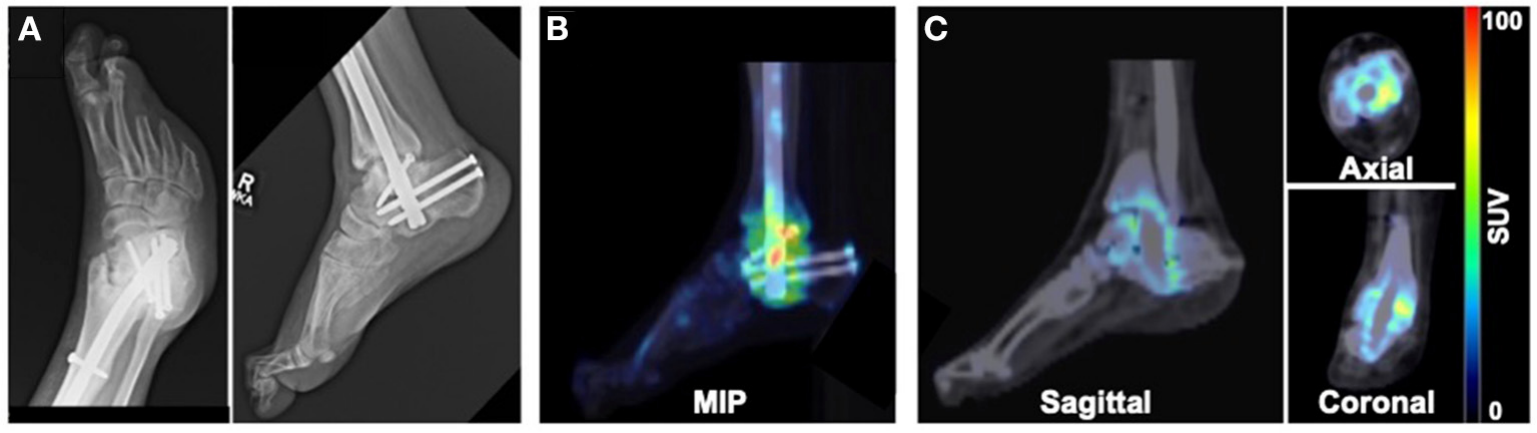
Imaging evaluation of 40-year old male patient with history of Charcot joint of the right ankle. **(A)** X-rays acquired 9 months after surgical reconstruction revealed stable bone architecture. **(B)** MIP and **(C)** PET/CT images acquired 9 months after surgery demonstrated focal uptake of ^18^F-NaF in the region of the surgically reconstructed ankle impacted by CN, suggesting ongoing remodeling of the bones of the ankle.

Patient 3 presented with a chronic wound on the plantar aspect of the right third digit as well as semi-rigid hammertoe contractures of digits 2 and 3 of the right foot, with the third digit ultimately undergoing amputation. The patient also had a history of Charcot joint of the right ankle and had intramedullary fixation applied to stabilize the ankle. X-rays acquired 9 months after surgical fixation showed stable bone structures without progression of the CN deformity (Figure 3A). PET/CT imaging at 9 months after surgical intervention revealed increased focal uptake of ^18^F-NaF at the level of the right ankle (Figures 3B,C), suggesting ongoing bone remodeling at the level of prior surgery.

**Figure 4:**
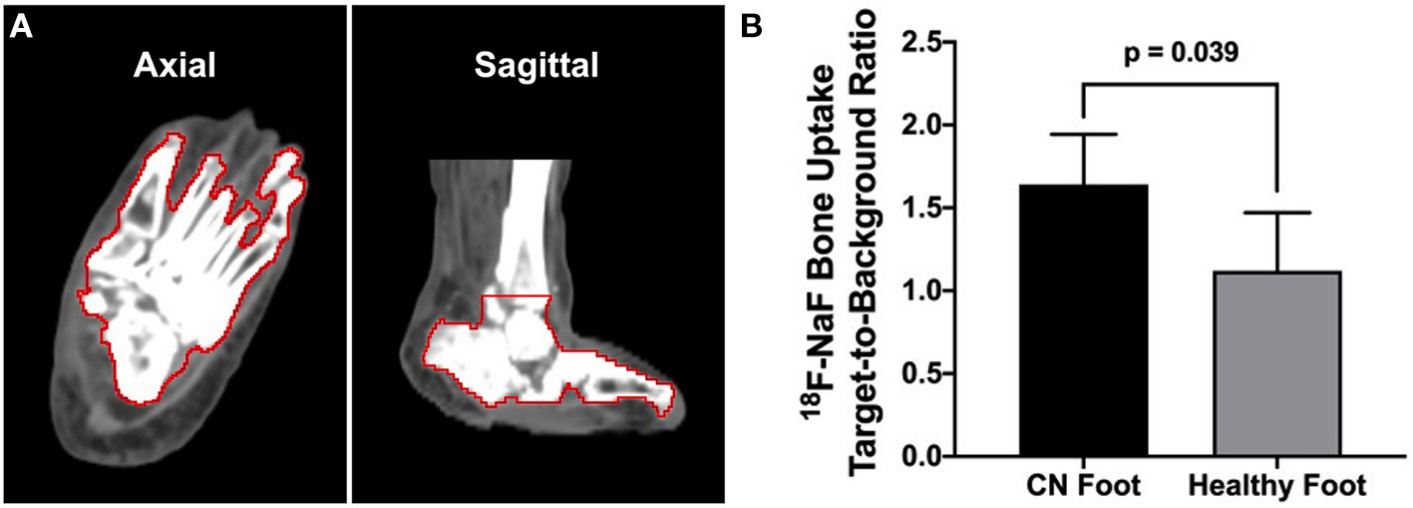
CT image-guided segmentation of the bones of the foot and quantitative ^18^F-NaF PET/CT image analysis. **(A)** Semiautomated segmentation of the bones of the foot and ankle (bone VOI outlined in red). **(B)** Quantitative analysis of PET/CT imaging demonstrated significantly higher bone uptake of ^18^F-NaF in the CN foot compared to the healthy non-CN foot. *N* = three patients. Values represent means ± SD.

Segmentation of the bones of the ankle and feet was achieved using our semiautomated CT image analysis approach (Figure 4A). Quantitative PET/CT image analysis demonstrated significantly higher ^18^F-NaF uptake (i.e., TBR) in the foot afflicted by CN compared to the healthy foot (CN foot: 1.64 ± 0.30 vs. healthy foot: 1.12 ± 0.35; *p* = 0.039) (Figure 4B).

## Discussion

The series of cases in the present study represent the first clinical application of ^18^F-NaF PET/CT imaging for assessing active bone remodeling in patients with CN. PET/CT imaging demonstrated increased retention of ^18^F-NaF in the lower extremity bones impacted by CN as early as 9 months and as late as 5 years following surgical reconstruction of the foot/ankle, thus suggesting that CN is a persistent condition characterized by active bone turnover that may not be fully suppressed for years after surgical intervention. Additionally, these initial cases reveal the potential of ^18^F-NaF PET/CT imaging for non-invasively detecting the active process of CN. By comparison, standard of care x-rays of the feet/ankles could not identify the continued neuroarthropathic process following surgical reconstruction.

Prior studies using nuclear medicine imaging approaches have primarily focused on assessing the inflammatory origins of CN or differentiating between soft tissue infection vs. osteomyelitis in the feet of patients with CN (25). In recent years, PET/CT imaging with ^18^F-FDG has emerged as a quantitative imaging approach for non-invasively evaluating the inflammatory origins of CN; however, ^18^F-FDG does not provide insight into the active process of bone remodeling. While ^99m^Tc-MDP does provide insight into active remodeling of bone, ^18^F-NaF has significantly more bone absorption than MDP. Additionally, PET imaging is more quantitative in nature and possesses higher spatial resolution than scintigraphy or SPECT, thereby offering potential advantages over conventional approaches for the evaluation of bone remodeling in patients with CN. Furthermore, due to its well-established use as a bone perfusion imaging radionuclide (21), future studies performing dynamic PET imaging at the time of ^18^F-NaF administration may provide additional functional assessment of the feet in patients with CN. The present investigation is the first imaging study to evaluate active bone remodeling in patients with CN using a quantitative PET/CT imaging method. This proof-of-concept report reveals that ^18^F-NaF PET/CT imaging may serve as a non-invasive biomarker for monitoring ongoing bone remodeling for months or years following surgical reconstruction of the foot in patients with CN. Additional work is needed to understand the potential role of ^18^F-NaF PET/CT imaging in the diagnosis and treatment planning for patients with CN.

## Data Availability Statement

The raw data supporting the conclusions of this article will be made available by the authors, without undue reservation.

## Ethics Statement

The studies involving human participants were reviewed and approved by the Institutional Review Board at Nationwide Children's Hospital. The patients/participants provided their written informed consent to participate in this study.

## Author Contributions

NT, T-HC, and MS were involved in the conception and design of the study. T-HC collected and organized the data. SP and LE assisted with patient enrollment and chart review. NT analyzed the imaging data and wrote the initial draft of the manuscript. MG, SA, and MS critically reviewed and assisted in the preparation of the manuscript. All authors made critical contributions to the manuscript, approved the final version of the manuscript, and took responsibility for the findings of the study.

## Funding

This work was supported by National Institutes of Health award R01 HL135103.

## Conflict of Interest

The authors declare that the research was conducted in the absence of any commercial or financial relationships that could be construed as a potential conflict of interest.

## Publisher's Note

All claims expressed in this article are solely those of the authors and do not necessarily represent those of their affiliated organizations, or those of the publisher, the editors and the reviewers. Any product that may be evaluated in this article, or claim that may be made by its manufacturer, is not guaranteed or endorsed by the publisher.
